# Exploring potential therapeutic targets for colorectal tumors based on whole genome sequencing of colorectal tumors and paracancerous tissues

**DOI:** 10.3389/fmolb.2025.1605117

**Published:** 2025-07-04

**Authors:** Yufan Sheng, Sen Niu, Da Li, Chunyuan Meng, Tong Wang

**Affiliations:** ^1^ Department of General Surgery, Wuxi People’s Hospital Affiliated to Nanjing Medical University, Wuxi, China; ^2^ Department of General Surgery, The First Affiliated Hospital of Nanjing Medical University, Nanjing, China

**Keywords:** colorectal cancer, whole genome sequencing, somatic mutation, germline mutation, cancer susceptibility gene, *SH3BP1*

## Abstract

**Objective:**

Colorectal cancer (CRC) is the third most common tumor worldwide and the second leading cause of cancer-related deaths. Colorectal cancer progresses slowly, and patients have to endure pain for a long time, both before and after surgery. The incidence rate has been increasing year by year in recent years, and the survival rate of patients is low. The number of new cases and deaths is expected to grow to 3.2 million and 1.6 million, respectively, by 2040. Currently, limited treatment (including surgical resection and radiotherapy) and systemic treatment (including chemotherapy and immune-targeted therapy) are the mainstays of colorectal cancer treatment, but the delay in treatment is still caused by untimely detection. In addition, some colorectal cancers are due to diet and lifestyle habits, but some are still due to heredity. Therefore, it is of great importance to analyze the genomic profiles of colorectal cancer and its paraneoplastic tissues, explore the disease-causing risk genes, and search for the potential novel therapeutic targets to improve the therapeutic efficacy of colorectal cancer.

**Methods:**

Tumor and adjacent tissue samples were harvested from 26 colorectal cancer patients and divided into tumor and paraneoplastic tissues. Whole genome sequencing was performed on these two groups of samples to obtain somatic and germ line mutation data of the two groups of samples, and then based on the data of the two groups, we screened and analyzed the mutation spectra and mutation characteristics, high-frequency copy number variations, and high-frequency mutated genes of tumor and paraneoplastic samples, thus mapping the genomic map of tumor and cancer susceptibility genes of paraneoplastic samples. The genomic profiles of the tumor and paraneoplastic samples were subsequently mapped. Finally, we performed pan-cancer expression analysis of *SH3BP1*, a susceptibility gene with a high mutation frequency. We also performed differential analysis of *SH3BP1* gene expression. We verified its expression level and function by protein blotting, immunohistochemistry, and cell scratch and cell viability assays. These assays further confirmed the validity and reliability of the sequence genome profiles and explored new therapeutic targets for colorectal cancer.

**Results:**

Significant gene mutation differences were observed between the tumor and adjacent normal tissue by whole genome sequencing. Gene spectral analysis of the tumors revealed that the tumors were characterized by C>T mutation types, and most of the samples were dominated by signature A mutation characteristics. High-frequency copy number analysis showed that most samples had increased copy numbers of gene fragments on chromosomes 7, 13, and 20. In addition, one high-frequency mutated gene (ATAD3B) and six candidate susceptibility genes were screened from the mutation data results, in which the probabilities of susceptibility genes mutated in normal tissues next to cancer were *CPA6* (3.85%), *ZNF888* (46.15%), *SH3BP1* (76.92%), *ANKRD16* (30.77%), *ATN1* (11.54%), and *C4orf54* (80.77%); based on which we roughly came up with more ideal cancer susceptibility genes, i.e., *SH3BP1* and *C4orf54.* Then, using *SH3BP1* as a target gene, we demonstrated increased *SH3BP1* expression in numerous cancers, particularly COAD and READ, through pan-cancer and differential gene expression analyses. We also verified differential *SH3BP1* expression in tumor and precancerous tissues using immunohistochemistry and protein blotting. After breaking down *SH3BP1* expression in cells, we performed a cell scratch assay. The cell scratch assay showed that tumor cell migration was reduced in cells with low *SH3BP1* expression. The CCK8 assay showed that tumor cell proliferation slowed down in cells with low *SH3BP1* expression compared to cells with high SH3BP1 expression. This suggests that *SH3BP1* may promote tumor proliferation and migration in colorectal cancer and offers the opportunity to be used as a therapeutic target.

**Conclusion:**

The large differences between single nucleotide mutations and insertion-deletion mutations in somatic cells and germ line cells indicate the large changes in the genome, especially in the non-coding region genome, during the transformation of normal tissue adjacent to cancer into cancerous tissue. In addition, the susceptibility gene *SH3BP1* found in this study has the role of promoting colorectal cancer, which has good research value and is expected to be a new target for colorectal cancer treatment.

## 1 Introduction

Colorectal cancer (CRC) is a common malignancy and a major global public health problem. (Abedizadeh et al., 2024). Colorectal cancer is known to be influenced by environmental and genetic factors, as well as unique molecular alterations, genomic manifestations, and pathogenesis. It shows obvious heterogeneity as well as genomic instability and molecular alterations, and this instability includes chromosomal instability and microsatellite instability, which can be categorized into four molecular types based on clinical and pathological features, including MSI (immune-type), CMS2 (classic-type), CMS3 (metabolic-type), and CMS4 (mesenchymal-type), and the different types have different clinical manifestations and different types have different clinical manifestations and prognostic relevance, requiring the development of targeted treatments (Guinney et al., 2015; Li J. et al., 2021; Golas et al., 2022; Dunne and Arends, 2024). Colorectal cancer can be divided into early-onset and late-onset; the probability of early-onset colorectal cancer in younger patients is increasing every year, and by around 2030, patients with early-onset colorectal cancer will account for 11% of the total number of colon cancers and 23% of the total number of rectal cancers, respectively (Akimoto et al., 2021; Spaander et al., 2023), and early-onset colorectal cancer has a higher mutation frequency and lower survival rate than other types (Ionescu et al., 2023). Therefore, mapping the colorectal cancer genome and searching for new potential therapeutic targets are of significant importance for clinical diagnosis and treatment.

Whole genome sequencing (WGS) can map a large number of genomic variants without the need for. Sequential genetic testing and has become an important tool for molecular genetic diagnosis of many diseases (Bagger et al., 2024). Whole genome sequencing has obvious advantages over other sequencing methods, as whole exome sequencing (WEG) is unable to sequence the non-coding regions, while whole genome sequencing is not only able to detect genes with variants in the non-coding regions but also able to target enough genes in the coding regions for better coverage (Kim et al., 2023). Whole genome sequencing is widely used, especially for the study of tumor genome can provide incomparable help (Meggendorfer et al., 2022). Whole genome sequencing is used in colorectal tumors and paracancerous tissues to analyze their genetic profiles, which can open a new direction for tumor treatment.

SH3 structural domain binding protein-1 (*SH3BP1*) is a member of the RhoGAP family (Schlam et al., 2015), which regulates the GTP-activating protein (GAP) activity of Rac1 by binding to the exocytic complex, thereby modulating cellular motility and migration functions (Parrini et al., 2011). In addition, *SH3BP1* acts as a GTPase-activating protein of Cdc42 and forms a complex with filamentous protein plus capsid protein (CapZ) to restrict Cdc42 signaling to regulate actin-driven cell membrane remodeling and intercellular junctions, maintains the morphology, stability, and connectivity of epithelial cells, and may be involved in epithelial-mesenchymal transition (EMT) (Elbediwy et al., 2012; Kang et al., 2020), and is thus considered an important regulator of cancer cell metastasis, which may promote the value-added migration of tumor cells. However, the expression and related roles of *SH3BP1* in colorectal cancer (CRC) have not been investigated. Therefore, as a susceptibility-pathogenicity gene screened in the genomic map of CRC, its role in CRC is worth exploring.

In this study, we used a combination of whole genome sequencing and basic experiments to map the genomic profiles of colorectal tumors and parasites tissues. We also explored the expression and function of the cancer susceptibility gene *SH3BP1* in colorectal cancer. Our results demonstrated significant differences in gene mutations, mutation frequency, mutation features, and copy number mutations between tumor and paraneoplastic tissues. We also screened the corresponding high-frequency mutated and susceptibility genes. First, pan-cancer expression analysis revealed that SH3BP1 is highly expressed in many cancers. Gene expression difference analysis immediately followed and proved that *SH3BP1* showed high expression in COAD and READ. Finally, experimental results showed that SH3BP1 was differentially expressed between colorectal tumors and normal tissues adjacent to cancer. *SH3BP1* also promoted tumor proliferation and migration. Thus, *SH3BP1* has the potential to be utilized as a therapeutic target for colorectal cancer and can provide new ideas for its treatment.

## 2 Materials and methods

### 2.1 Patient and tissue samples

Tumor specimens and corresponding precancerous normal tissues were collected from 26 colorectal cancer patients from October 2023 to January 2024, excluding patients with advanced disease, active malignancies, or those who had received radiotherapy or chemotherapy. All tissue specimens were available from patients diagnosed with colorectal cancer by histopathological examination at Wuxi People’s Hospital, and all specimens were frozen in liquid nitrogen and transferred to the specimen bank of the Clinical Research Center. 26 patients were identified by their sample name, sample type, sampling time, sex, age, height, weight, differentiation grade, TNM staging, pathology, clinical staging, immunohistochemistry, tumor size, vascular invasion, and nerve invasion, as described in the following table. Invasion, and nerve invasion, as detailed in Supplementary Table S1. This study was conducted in accordance with the ethical standards of Helsinki, and the Medical Ethics Committee of Wuxi People’s Hospital approved the study.

### 2.2 DNA extraction and quality control

Sample DNA was extracted using the TIANNamp Genomic DNA Kit (Tiangen Biotech, China), and DNA quality control was performed using the following two methods: Nanodrop for DNA concentration and Agilent 4150/5400 for DNA integrity.

### 2.3 Library building and sequencing

Using the IGT Enzyme Plus Library Prep Kit V3 (iGeneTech, China), the DNA was cut into fragments of approximately 350 bp, end repaired, “A” tailed, and annealed to the sequencing junctions, and then the products were purified by magnetic beads. Then the product was purified by magnetic beads, and the original library with moderate fragment length was selected for the next step of PCR amplification. The amplified product was purified by magnetic beads, and the small fragment library for sequencing was obtained.

After the library construction was completed, Qubit was used for quantification, followed by Agilent 4150/Qseq400 to test the insert size of the library to ensure its quality. Sequencing was performed on an Illumina Noveaseq 6000 (Kokuryo et al., 2025). The sequencing mode was PE150, which means that in the constructed DNA small fragment library, each insert fragment was sequenced at both ends, 150 bp at each end (Li X. et al., 2021).

### 2.4 Data quality control

Data filtering primarily eliminates the following three situations: the length after truncation of the splice sequence is less than 50 bpm; the number of bases at one end of a single piece of data is more than 5% of the total base ratio of that piece of data; and the number of inferior bases at one end of a single piece of data is more than 50% of the total bases of that piece of data.

### 2.5 Compare data

The filtered data were compared to the human reference genome using the Burrows-Wheeler alignment tool (BWA) (Li and Durbin, 2009). The initial results in BAM format were obtained after BMA processing, and the final comparison results in BAM format were obtained by further using Picard software for processing, such as labeling repetitive sequences. These aligned results were used to perform statistics on sequencing depth and coverage.

### 2.6 Somatic and germline mutations

GATK-Mutect2 was used to detect somatic single nucleotide mutations, insertion and deletion mutations (Pei et al., 2021), and these sequences were compared and corrected with paracancerous samples to filter out normal tissue information. The tested somatic mutation data were annotated using ANNOVAR software. Detection of germline single nucleotide mutations and insertion and deletion mutations was performed using GATK.

### 2.7 Mutation spectrum and mutation characterization, high-frequency mutant gene analysis, high-frequency single nucleotide variant analysis

Single nucleotide mutations based on somatic cells were analyzed from multiple perspectives, and the mutation spectrum and mutation characteristics of the derived tumor samples were plotted using R language software. Fisher’s exact test method was used to screen for high-frequency mutated genes in the somatic cell mutation data (Mateo et al., 2020). The distribution of copy number mutations in the samples as well as on the chromosomes was analyzed using GISTIC software along with somatic single nucleotide mutation analysis.

### 2.8 Susceptibility screening

This genetic screen is based on the screening of paraneoplastic normal tissue germline variation data to derive susceptibility genes in paraneoplastic normal tissues that may lead to the transformation of carcinoma into cancer. The screening methods include (1) removing variants with sequencing depth less than 10X; (2) removing variant loci with a high frequency of Yubo 0.0014 in the Thousands, ExAc, and esp6500 databases, directly removing loci in the dbSNP database, and retaining loci in the COSMIC database; (3) deleting variant loci in intergenic, non-coding, and intronic regions; (4) filtering out synonymous mutations as well as phase duplication genes; and (5) removing harmless mutations according to the results of the ljb23-sift, ljb23-pp2hvar, ljb23-pp2hdiv, and ljb23-mt databases.

### 2.9 Data acquisition

Gene expression data were available from The Cancer Genome Atlas (TCGA) and the Genotype-Tissue Expression Project (GTEx) databases. This included RNA-seq data of *SH3BP1* from 33 types of cancer, as well as normal colorectal tissue. The data was normalized using the log2 (TPM+1) method and then employed to subsequent analysis. The results of the analyses were visualized using public R package.

### 2.10 Pan-cancer expression analysis

Duplicate and missing RNA-seq data from the GTEx and TCGA databases were exempted. The expression levels of SH3BP1 in various cancer types were tested and visualized in R Studio (version 4.2.3). Statistical comparisons were carried out using the “stats” (version 4.2.1) and “car” (version 3.1.0) packages. We chose analysis of variance (ANOVA) as the statistical method for assessing differential expression of SH3BP1. The analysis primarily examined differences in *SH3BP1* expression between various cancer and normal tissues.

### 2.11 Differential expression analysis

We assessed the expression levels of *SH3BP1* mRNA in colon (COAD) and rectal (READ) adenocarcinomas during gene expression comparisons between cancerous and normal tissues. Box plots were subsequently generated. Meanwhile, the expression levels of cancer susceptibility genes obtained from sequencing were compared in COAD and READ, and heat maps were created.

### 2.12 Cell lines and cell culture

Two human colorectal cancer cell lines (HCT116, RKO) and one normal human intestinal epithelial cell line (NCM460) were employed in this experiment. All cells were available from the Clinical Research Center of Wuxi Medical Center, Nanjing Medical University. All cells were grown in DMEM medium (Gibco, United States) containing fetal bovine serum (Biological Industries, Israel) and double antibody (NCM Biotech, China). They were cultured at 37°C in a constant temperature incubator containing 5% carbon dioxide.

### 2.13 Western blot

RIPA lysis buffer (CWBIO, China) was mixed with EDTA, protease inhibitor, and phosphatase inhibitor (Beyotime, China) at a ratio of 1:100 (1000µlRIPA, 10µlEDTA, 10 µL protease inhibitor, 10 µL phosphatase inhibitor) to lose the cells and allowed to stand on ice for 20 min. The cells were centrifuged at 12,000 rpm for 10 min, and the supernatant was combined with loading buffer (CWBIO, China) at a ratio of 4:1. The mixture was boiled for 5 min, removed, and permitted to stand at room temperature before sodium dodecyl sulfate-polyacrylamide gel electrophoresis (SDS-PAGE). The protein was then transferred to a polypropylene fluoride membrane (PVDF, Millipore, United States) by membrane transfer, followed by rapid sealing with sealing solution (Beyotime, China) for 30 min, and then placed in the box with diluted primary antibodies *SH3BP1* (1:5000, Proteintech, China) and *GAPDH* (1:2000, Affinity Biosciences, United States) overnight at 4°C in a refrigerator shaker. The next day, the antibody was retrieved, washed three times with TBS (Tris-HCl buffered saline) and Tween (TBST, CWBIO, China), diluted with secondary antibody (1:2000, Proteintech, China), and shaken for 1 h, and then washed three times with TBST. The 1:1 developer (ThermoFisher, United States) was exposed. Data quantification was conducted using the Fiji software (ImageJ, United States). *GAPDH* served as a control in this study.

### 2.14 Immunohistochemistry

Tissue was trimmed, fixed in 10% paraformaldehyde for 24 h, dehydrated, cleared, embedded in wax, embedded, and cut into 4-μm thick sections. The sections were baked, delayed, hydrated, antigen retrieved, and blocked with endogenous peroxidase. The sections were subsequently incubated with 4% rabbit serum for 15 min at room temperature to reduce specific staining. The sections were subsequently incubated with primary antibody (1:500, Proteintech, China) for 2 h. This was accompanied by incubation with secondary antibody at room temperature for 30 min. Next, DAB was added dropwise for color development, monitored by staining with hematoxylin for 30 s, differentiation with 0.1% hydrochloric acid, rinsing with tap water for 3 min, blueing with ammonia, and further rinsing with tap water for 3 min. Finally, the slides were dehydrated, made transparent, and sealed. The slides were celebrated under a fluorescence microscope (ZEISS, Germany).

### 2.15 Transient transfection of cells

Cellular SH3BP1 expression was silenced by small interfering RNA (RIBOBIO, China). The sequences of shRNAs are the following.

si*SH3BP1*-1:5′-CCAGCAACATCGCCATAGT-3';
si*SH3BP1-*2:5′-GTCACAGCCATACGACCAT-3′
si*SH3BP1*-3:5′TGCCAGCCATCCTCAAACA-3'.

Opti-MEM medium (Gibco, United States) 125 µL was used to add to tubes containing 10 µL siRNA and lipo3000 (ThermoFisher, United States), respectively; then siRNA dilution was added to lipo3000 dilution, mixed well, and allowed to stand for 15 min. Transfection reagent was prepared for transfection.

### 2.16 Scratch test

Before the cells were passaged into the six-well plate, the scratch film (Beyotime, China) was placed in the plate, and UV light irradiation was applied for 20 min to start the passage and spreading of the plate, and the length was up to 90%. On the back of the six-well plate, two horizontal lines were drawn along the ruler as a marking line to observe the cells; the original culture medium was discarded, the film was gently torn off, and the scratch film intersected with the marking line perpendicularly, and the intersection point was used as a fixed detection point. Immediately after serum-free DMEM medium (Gibco, United States) was added, the cell photographs were observed with a microscope (Nikon, Japan) as a control and then placed in an incubator, and the growth of cells near the scratches was recorded at different times: 0 h, 24 h, and 48 h.

### 2.17 Cell viability assay (CCK-8)

RKO and HCT116 colorectal cancer cells, which were transformed with either si-NC or si-*SH3BP1*, were inoculated into 96-well plates at a density of 2,000 cells per well. Three groups were established: experimental, control, and blank, with six replicates in each group. At 0, 1, 2, 3, 4and 5days, 10 µL of CCK-8 reagent was in addition to each well. The wells were not subject to light and incubated for 2 hours in an incubator. Measure the optical density (OD) at 450 nm using a spectrophotometer. Generate a growth curve.

### 2.18 Statistical analysis

All data are expressed as mean ± SEM (±SD). Analytical plotting for this postgraduate letter section was performed by R4.2.3. Descriptive statistics were utilized to summarize sample data characteristics as well as mutation frequencies, while Fisher’s exact test was applied. All raw p values for the above tests were adapted by a very conservative Bonferroni correction. Images were analyzed and processed in the experimental part using Fiji software (ImageJ, United States) and GraphPad Prism 10.1.2 (GraphPad Software, United States). All p-values <0.05 were examined statistically significant.

## 3 Result

### 3.1 Samples and clinical data

We performed whole-genome sequencing of colorectal tumors and their paracancerous normal tissues, and collected samples of tumors and their paracancerous normal tissues from 26 patients diagnosed with colorectal cancer; all samples were collected with the approval of the institutional ethical review board. Supplementary Tables S1 and Table 1 show the relevant clinical characteristics of the group. At the time of diagnosis, 42.31% of the patients were females and another 57.69% were male. Overall, 65.38% of patients in this cohort had adenocarcinoma, 7.69% and 3.85% had mucinous and tubular adenocarcinoma, respectively, and 19.23% had specific types of adenocarcinoma, including adenocarcinoma with mucinous adenocarcinoma and tubular-papillary adenocarcinoma. Vascular invasion and nerve invasion were found in 34.62% and 38.46% of patients, respectively. The percentages of patients classified as having clinical stage I, II, III, and IV disease were 7.69%, 30.77%, 61.54%, and 0%, respectively. In addition, 76.92% of the tumors had grade 2 differentiation, 19.23% had grade 2–3 as well as 3.85% had grade 3.

**TABLE 1:** Clinical characteristics of patients with colorectal cancer.

Features	Total (N = 26)
Cancer type, n (%)	
Adenocarcinoma	17 (65.38%)
Mucinous adenocarcinoma	2 (7.69%)
tubular adenocarcinoma	1 (3.85%)
Special types of adenocarcinoma	5 (19.23%)
Gender, n (%)	
Female	11 (42.31%)
male	15 (57.69%)
Height (mean ± SD)	159 ± 4.69
Weight (mean ± SD)	60.5 ± 3.70
Clinical staging, n (%)	
I	2 (7.69%)
II	8 (30.77%)
III	16 (61.54%)
IV	0 (0%)
pathological differentiation grade, n (%)	
Level 2	20 (76.92%)
Level 2–3 Level 3	5 (19.23%) 1 (3.85%)
Invasion of the vascular vessels, n (%)	
Ture	9 (34.62%)
false	17 (65.38%)
Nerve invasion, n (%)	
Ture	10 (38.46%)
false	16 (61.54%)

### 3.2 Genomic landscape of tumor samples

#### 3.2.1 Single nucleotide somatic mutation

Single nucleotide variants (SNVs) are caused by single nucleotide changes in the genome and are also known as point mutations. We performed whole genome sequencing on 26 colorectal cancer tumor samples and used GATK-Mutect2 to detect single nucleotide variants. As shown in Table 2, a total of 19,314 coding region exonic mutations, 7,102 synonymous mutations, 11,539 non-synonymous mutations, 105,9953 intergenic mutations, 63,4314 intronic mutations, and 188,7502 total mutations were detected. In colorectal cancer patients, the number of mutations occurring in exons outside the coding region accounts for approximately 10% or less of the total number of mutations, but there are three samples (E1, R1, and AF1) with particularly high numbers of mutations (1947, 7144, and 1467, respectively), which is also the case for synonymous mutations, coding region intronic mutations, and nonsynonymous mutations (Table 2). Single nucleotide mutations in CRC occur mainly in the intergenic region, followed by the intron region. Among the non-coding regions, there were a total of 8,406 exon mutations, while there were as many as 119,281 intron mutations, showing that intron mutations still predominate in the non-coding regions of CRC (Supplementary Table S2). The mutation rates of E1, R1, and AF1 in the non-coding regions remain high.

**TABLE 2:** Single nucleotide mutation statistics in the coding region of somatic cells.

Samples	Exonic	synonymous_SNV	nonsynonymous_SNV	Stopgain	Intronic	Total	Intergenic
A1	227	68	152	7	6252	21,306	12,920
G1	249	72	158	15	6019	20,090	11,927
B1	414	132	258	22	7832	24,640	14,052
D1	533	247	262	23	8719	27,061	15,300
F1	619	179	409	25	12,160	35,752	19,487
I1	545	173	336	31	11,315	36,516	21,284
K1	386	106	261	14	9870	33,730	20,316
L1	433	120	288	23	9793	37,074	18,094
E1	1947	558	1,318	61	41,822	113,951	59,500
M1	376	105	255	12	8755	29,232	17,438
N1	228	72	139	14	5785	19,622	11,757
O1	535	137	382	12	5162	16,082	8691
P1	279	80	186	10	6911	22,208	12,907
Q1	248	69	159	18	6352	22,205	13,498
R1	7,144	3507	3,521	54	376,249	1,072,787	591,740
S1	406	133	270	20	10,539	35,962	21,713
V1	290	76	197	15	6,581	20,391	11,633
X1	264	98	160	3	6,760	21,771	12,673
Y1	380	114	248	17	10,885	33,840	19,551
Z1	452	119	300	13	12,378	39,770	23,283
AA1	438	109	298	19	9,534	31,315	18,500
AB1	309	139	187	7	8,615	30,038	18,289
AC1	473	111	318	13	12,009	38,136	22,047
AD1	325	104	196	14	7,742	25,855	15,368
AE1	347	142	219	20	9,293	30,350	17,967
AF1	1,467	362	1,060	27	16,982	53,818	30,044

Exonic: The number of mutations that occur in the exon coding region; synonymous_SNV: number of synonymous mutations; nonsynonymous_SNV, Number of non-synonymous mutations; stopgain:The number of nonsense mutations that result in the production of a new stop codon; intronic: The number of mutations that occur in a gene’s intron; intergenic: The number of mutations occurring in the intergenic region; total: Total number of single nucleotide mutations (SNV).

#### 3.2.2 Somatic insertion and deletion mutations

Based on somatic mutations, we continued to use GATK-Mutect2 to detect insertion and deletion (Indel) mutations. Insertion and deletion mutations in CRC occur in coding regions at the same site as single nucleotide mutations, mainly in intergenic regions and to a lesser extent in intronic regions (Tables 2, 3). Insertions and deletions that occur in the coding region or splice site of the genome are more likely to be dramatically alter the translation of the protein. This is called a frameshift mutation, and the length of the base pairs of the insertion (frameshift_insertion) and deletion (frameshift_deletion) is usually a non-integral multiple of 3, which can lead to a change in the entire reading frame, which in turn affects protein synthesis. Compared to non-frameshift mutations, frameshift mutations have a greater impact on gene function. As can be seen in Table 3, the number of mutations that have occurred in the E1 and R1 samples is the most obvious, with the E1 having more insertion mutations, about 0.0147% of the total, and the other having more deletion mutations, about 0.036% of the total. This implies that the proteins in these three cancer tissue samples have undergone fundamental changes. Insertion and deletion mutations in non-coding regions There were 1164 exon mutations, 28,980 intron mutations, and 9 mutations at cleavage sites, which was the same as single nucleotide mutations, with intron mutations being the main type (Supplementary Table S2; Supplementary Table S3). Among the few cleavage site mutations, E1 and R1 account for 33.33% each. As mentioned above, cleavage site mutations can change the reading frame of protein translation, which means that gene mutations in E1 and R1, whether in the coding or non-coding region, can profoundly affect the expression of tissue proteins (Supplementary Table S3).

**TABLE 3:** Statistical table of insertions and deletions of coding regions in somatic cells.

Samples	Exonic	frameshift_deletion	frameshift_insertion	Intronic	Intergenic	Total
A1	18	13	1	2,772	3,371	6,783
G1	13	2	2	2,117	2,660	5,260
B1	15	8	4	1,892	2,345	4,692
D1	17	9	3	2,374	3,005	5,916
F1	9	6	0	3,048	3,703	7,453
I1	23	21	0	3,213	4,210	8,183
K1	9	7	2	2,350	3,076	5,972
L1	16	6	3	2,676	3,756	7,048
E1	366	25	73	73,444	80,699	169,616
M1	27	18	3	3,089	4,134	7,962
N1	13	9	2	3,054	3,619	7,315
O1	21	18	1	1,540	1,813	3,775
P1	9	8	0	3,274	3,922	7,875
Q1	15	8	3	2,375	3,055	5,975
R1	184	44	21	45,941	65,547	123,671
S1	19	11	4	4,610	3,710	12,466
V1	14	11	0	2,772	3,102	6,469
X1	5	2	0	2,641	3,179	6,400
Y1	21	14	2	3,515	4,436	8,771
Z1	15	7	4	4,173	5,993	11,125
AA1	21	14	3	2,827	3,556	7,033
AB1	21	15	3	3,027	4,112	7,800
AC1	26	18	2	3,692	4,818	9,361
AD1	20	16	2	2,804	3,375	6,785
AE1	13	8	2	3,167	3,922	7,768
AF1	41	22	2	2,679	3,637	6,937

Exonic: The number of mutations that occur in the exon coding region; frameshift_deletion: The number of frameshift deletions, an integer multiple of the deletion length of 3, resulting in a change in the reading frame of the encoded protein; frameshift_insertion: The number of frameshift insertions, an integer multiple of the insertion length of 3, resulting in a change in the reading frame of the encoded protein; intronic: The number of mutations that occur in a gene’s intron; intergenic: The number of mutations occurring in the intergenic region; total: Total number of mutations.

### 3.3 Genomic landscape of samples adjacent to cancer

#### 3.3.1 Single nucleotide mutation in germline

Germline mutations are mutations carried during embryonic development. They are highly heritable and make up the genetic diversity between people. All cells in the human body carry the same germline mutations. To obtain information about germline single nucleotide variants, the GATK software was used to detect germline mutations in the tissue adjacent to the cancer. In the coding region, we found 554,590 exonic mutations, 285,765 synonymous mutations, 260,333 non-synonymous mutations, 329,132,950 intronic mutations, 541,376,470 intergenic mutations, and a total of 961,379 52 (Table 4). Unlike somatic single nucleotide mutations in coding regions, germline single nucleotide mutations did not differ significantly between samples. However, the similarity is that single nucleotide mutations are still mainly distributed in the intergenic region, followed by the intronic region (Tables 2 and 4). In addition, as indicated in Supplementary Table S4, we detected 370,442 exon mutations, 5,956,492 intron mutations, and 1,925 cleavage site mutations in non-coding regions. Compared to the mutation situation in the non-coding region of somatic single nucleotide mutations, the mutation distribution is relatively balanced (Supplementary Table S2; Supplementary Table S4).

**TABLE 4:** Statistics of single nucleotide mutations in coding regions of germline cells.

Samples	Exonic	synonymous_SNV	nonsynonymous_SNV	Intronic	Intergenic	Total
A2	21,330	10,965	10,062	1,236,460	1,995,044	3,571,762
G2	20,760	10,795	9,714	1,204,640	1,954,451	3,489,991
B2	21,492	10,999	10,128	1,279,819	2,092,707	3,724,867
D2	21,420	10,962	10,086	1,270,916	2,102,319	3,733,156
F2	21,249	10,972	9,929	1,268,598	2,092,974	3,712,288
I2	21,418	10,998	10,072	1,264,252	2,091,372	3,706,014
K2	21,218	10,928	9,956	1,270,104	2,102,216	3,724,556
L2	21,436	10,991	10,114	1,247,137	2,084,863	3,712,818
E2	21,036	10,875	9,861	1,268,017	2,080,251	3696616
M2	21,042	11,073	10,013	1,270,706	2,116,131	3736293
N2	21,348	10,944	10,015	1,277,324	2,113,961	3,744,350
O2	21,283	10,961	10,058	1,269,724	2,111,857	3,734,106
P2	21,368	11,002	9,844	1,265,521	2,087,451	3,703,209
Q2	21,185	10,963	9,986	1,261,794	2,080,751	3,690,318
R2	21,289	11,000	10,062	1,274,074	2,103,933	3,731,153
S2	21,380	11,115	10,129	1,271,932	2,107,524	3,731,078
V2	21,578	10,682	9,703	1,250,046	1,952,921	3,545,933
X2	20,709	11,186	10,118	1,281,237	2,093,248	3,727,021
Y2	21,640	11,047	9,991	1,276,007	2,089,311	3,715,939
Z2	21,372	11,054	10,066	1,274,270	2,096,588	3,721,788
AA2	21,450	11,164	10,071	1,268,904	2,094,797	3,714,931
AB2	21,608	10,974	10,054	1,268,904	2,098,742	3,714,117
AC2	21,392	10,989	10,050	1,267,919	2,092,248	3,707,441
AD2	21,357	11,192	10,274	1,265,748	2,104,546	3,730,655
AE2	21,696	10,960	10,029	1,274,676	2,104,325	3,716,033
AF2	21,280	10,974	9,948	1,262,132	2,089,116	3,701,519

Exonic: The number of mutations that occur in the exon coding region; synonymous_SNV: number of synonymous mutations; nonsynonymous_SNV, Number of non-synonymous mutations; intronic: The number of mutations that occur in a gene’s intron; intergenic: The number of mutations occurring in the intergenic region; total: Total number of single nucleotide mutations (SNV).

#### 3.3.2 Germline mutations insertions and deletions

Immediately above, while using the GATK software to detect germ line mutations in normal tissue adjacent to cancer, we also obtained data on germ line insertion and deletion mutations. A total of 14,588 coding region exon mutations, 2,468 synonymous mutations, 1,629 non-synonymous mutations, 99,223,250 coding region intron mutations, 131,253,980 intergenic mutations, 57,200 non-coding region exon mutations, 15,803,140 non-coding region intron mutations, 425 non-coding region breakpoints, and 25,373,089 total mutations (Table 5; Supplementary Table S5). Comparing the single nucleotide mutations and insertions and deletions in germ line cells (Table 4; Table 5; Supplementary Table S4; Supplementary Table S5) with the somatic mutations (Table 2; Table 3; Supplementary Table S2; Supplementary Table S3), it was found that the number of germ line mutations was always higher than that of somatic mutations, and the mutation changes were mainly concentrated in non-coding regions, with individual samples showing a change of nearly 100-fold. In addition, there was no variation in germ line mutations between samples as there was in somatic cells. This implies that during the transformation of normal cells adjacent to cancer cells into cancer cells, there are significant changes in the genome in both coding and non-coding regions, with particularly drastic changes occurring in non-coding regions.

**TABLE 5:** Insertion and deletion statistics in coding regions of germline cells.

Samples	Exonic	Frameshiftdeletion	Frameshiftinsertion	Intronic	Intergenic	Total
A2	565	99	59	355,446	460,273	898,904
G2	523	106	57	356,572	458,147	896,558
B2	572	89	65	399,575	528,756	1021744
D2	561	94	60	400,081	532,223	1026546
F2	577	96	66	394,956	525,037	1012445
I2	527	92	68	384,517	511,058	985,970
K2	535	91	56	386,048	515,689	992,875
L2	544	93	64	388,004	513,220	992,320
E2	571	98	60	371,785	493,067	952,055
M2	587	100	74	385,958	510,992	987,349
N2	581	106	61	378,004	504,409	971,206
O2	572	100	64	392,786	521,801	100,6409
P2	523	78	50	368,616	492,902	948,192
Q2	563	101	60	375,047	496,754	959,325
R2	561	86	63	383,477	507,169	980,824
S2	582	97	71	379,755	506,167	975,154
V2	512	82	56	373,290	480,130	941,224
X2	541	78	64	383,130	504,031	976,614
Y2	561	86	61	398,658	524,861	1016640
Z2	564	93	59	385,988	511,170	987,518
AA2	578	92	67	381,971	504,687	976,361
AB2	563	102	65	373,469	497,559	958,528
AC2	566	103	65	380,856	505,078	975,331
AD2	591	105	65	380,984	505,687	975,705
AE2	580	103	63	381,219	509,810	980,485
AF2	588	98	66	382,133	504,721	976,807

Exonic: The number of mutations that occur in the exon coding region; frameshift_deletion: The number of frameshift deletions, an integer multiple of the deletion length of 3, resulting in a change in the reading frame of the encoded protein; frameshift_insertion: The number of frameshift insertions, an integer multiple of the insertion length of 3, resulting in a change in the reading frame of the encoded protein; intronic: The number of mutations that occur in a gene’s intron; intergenic: The number of mutations occurring in the intergenic region; total: Total number of mutations.

### 3.4 Mutation spectrum and mutation signature

Multidimensional analysis of somatic single nucleotide mutations (point mutations), including mutation spectrum and mutation signature. From these results, we can clearly understand the characteristics of cancer mutations at the point mutation level, i.e., at the base level.

#### 3.4.1 Mutation spectrum analysis

**FIGURE 1:**
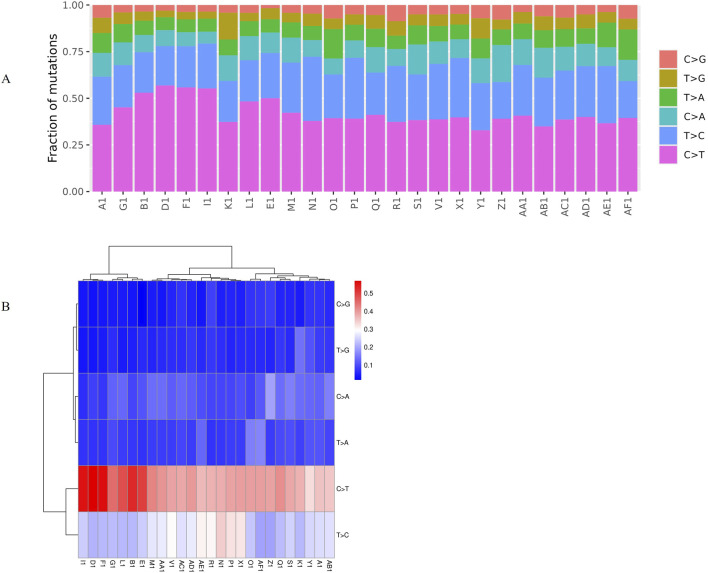
A bar chart and heat map of the mutation spectrum of somatic single nucleotide mutations. **(A)** A bar chart of the mutation spectrum, where the abscissa represents different samples and the ordinate represents the proportion of mutation types, and different colors represent different mutation types. **(B)** A heat map of the mutation spectrum, where the abscissa represents different samples and the ordinate represents the mutation type, and the redder the color, the higher the proportion of the corresponding mutation type in the sample, and the bluer the color, the lower the proportion.

There are six types of variation in single base substitutions: C>A/G>T, C>G/G>C, C>T/G>A, T>A/A>T, T>C/A>G, and T>G/A>C (for simplicity, we will use C>A, C>G, C>T, T>A, T>C, and T>G, respectively). And can be divided into two categories according to the type of base that is replaced: a transversion occurs when a purine is replaced by a pyrimidine; a transition occurs when a purine is replaced by a purine or a pyrimidine is replaced by a pyrimidine. In each sample, the C>T mutation type is predominant, mainly base transitions between genomes, followed by T>C, C>A, T>A, T>G, and C>G in decreasing order (Figure 1A). In addition, the probability of base substitution in tumor samples is relatively low (Figure 1A). The mutation spectrum heat map also shows that the C>T mutation type accounts for a high proportion of each sample, especially B1, D1, E1, F1, and I1 (Figure 1B).

#### 3.4.2 Mutation signature analysis

**FIGURE 2:**
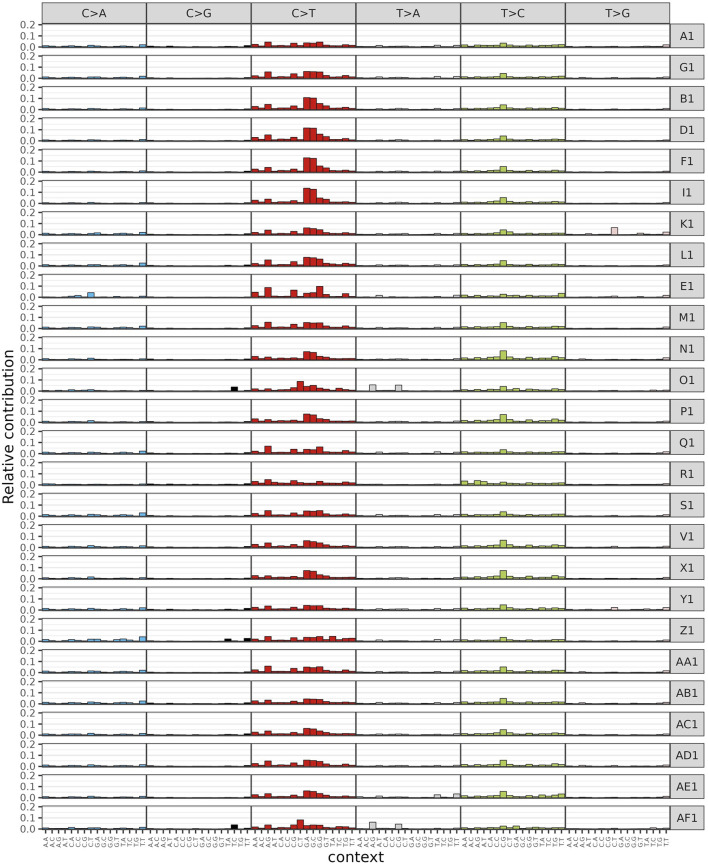
Shows a histogram of the frequencies of the 96 mutation types found in each sample. The horizontal axis represents the 96 mutation types, and the vertical axis represents the frequency of each mutation type in a sample. The higher the frequency, the more common the mutation type is in the sample. Different colors represent different mutation types.

**FIGURE 3:**
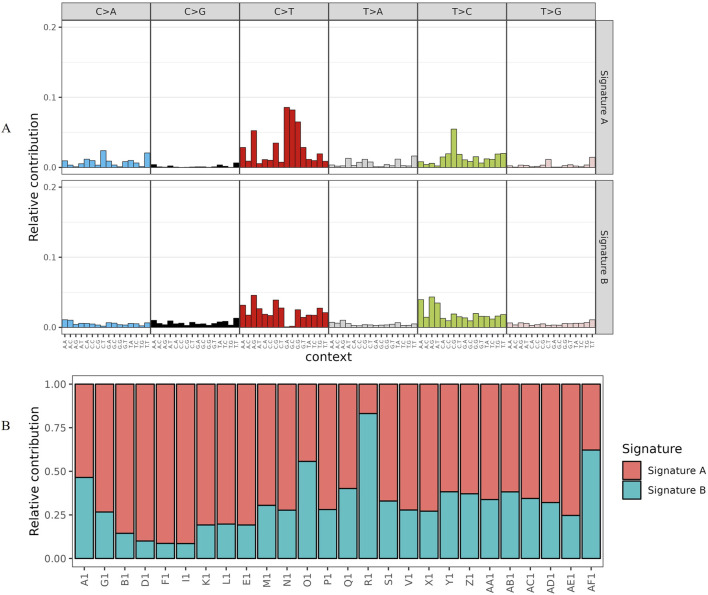
Histogram of mutation feature frequencies and contributions. **(A)** Shows the histogram of mutation feature frequencies. The horizontal coordinates represent the 96 mutation types, and the vertical coordinates represent the frequencies of the different mutation feature types. The higher the frequency, the higher the mutation frequency in the sample. **(B)** A plot showing the proportion of each mutation feature in different samples. The horizontal axis represents the samples, and the vertical axis represents the proportion of each mutation feature. The larger the proportion, the more prevalent the mutation feature is in the sample. Different colors represent different mutation features.

In the process of DNA replication, mismatches, induction by endogenous or exogenous mutagens, and defects in DNA repair mechanisms inevitably lead to somatic mutations; different mutation processes result in specific combinations of mutation types, referred to as mutation characteristics. By considering the base types at 1 bp upstream and 1 bp downstream of a single point mutation site, we have classified point mutations into 96 mutation types characterized by two combinations of bases, e.g., A-A, A-G, A-C, A-T, C-A, C-G, C-C, C-T, and so on. Tumor point mutation types were still found to be predominantly C>T, but specific mutation types were considered to be predominantly C-G, C-T, G-A, G-C, and G-G (Figure 2). Based on the mutation distribution of the 96 mutation types in each sample (Figure 2), the mutation characteristics of all samples were categorized into signature A and B. As shown in Figure 3A, the biggest difference between signature A and B is whether the difference between the specific 96 point mutation types of C>T and T>A is obvious. In signature A, the mutation types are dominated by G-A, G-G, G-C, and G-T. In signature B, the mutation types shift to be most significant in A-G, and C-G. In the mutation feature contribution graph, we can see that most samples are dominated by signature A mutation features, i.e., dominated by G-A, G-G, G-C, and G-T, and only individual samples such as R1 and AF1 are dominated by signature B mutation features (Figure 3B). At this point, we derived information about the mutation spectrum and mutation features of the complete tumor samples.

### 3.5 High-frequency copy number mutation analysis

**FIGURE 4:**
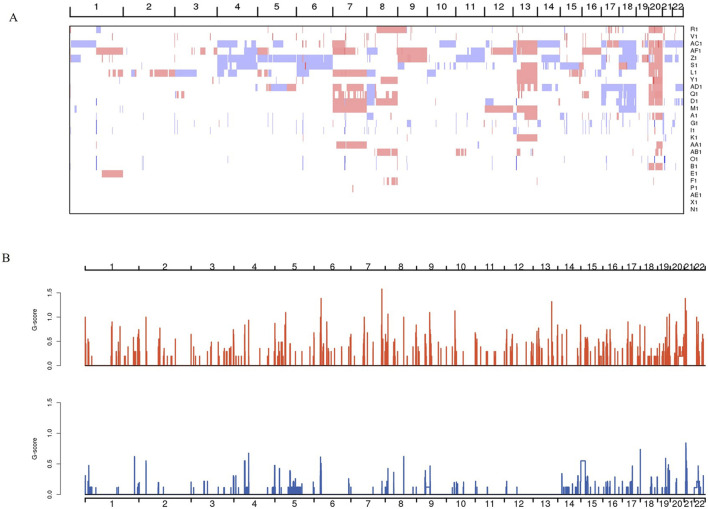
Somatic high-frequency copy number mutation analysis includes the distribution of copy number mutations in tumor samples and the distribution of high-frequency copy number mutations. **(A)** The distribution of copy number mutations in tumor samples is shown in the figure. The abscissa is chromosomes 1–22, and the ordinate is different samples. The red and blue parts of the figure are somatic copy number mutations in individuals, where red represents an increase in copy number and blue represents a decrease in copy number. The darker the color, the greater the copy number change. **(B)** A distribution map of high-frequency copy number mutations, with chromosomes 1–22 on the horizontal axis (sex chromosomes not considered) and the GISTIC software score for high-frequency copy number mutation segments on the vertical axis. A higher score indicates that the copy number mutation is more common in that segment. Red indicates copy number gain and blue indicates copy number loss.

Copy number variation (CNV) is an important part of genomic structural variation (SV), which can be categorized into two types: deletions and duplications. In the distribution map of copy number mutations in tumor samples, most of the samples showed copy number gains on chromosomes 7, 13, and 20, and mainly copy number losses on chromosomes 4, 6, 17, and 18 (Figure 4A). Meanwhile, in the high-frequency copy number mutation distribution map, we used the GISTIC software to score the high-frequency copy number mutation region; the higher the score, the higher the frequency of copy number mutation in this region, and we found that the copy number increase was mainly shown on chromosomes 1, 2, 3, 6, 7, 13, and 20, and the copy number decrease was mainly shown on chromosomes 4, 6, 17, 18, and 21 (Figure 4B). This indicates that the genomes of tumor samples show different alterations of gene fragments on different chromosomes, which is more favorable evidence that tumors are caused by a series of mutations or aberrations accumulated at the level of somatic genome, and that genomic alterations are crucial in the process of tumorigenesis and development.

### 3.6 Significantly mutated gene analysis

Significantly mutated genes (SMGs) include somatic single nucleotide mutations and insertion/deletion mutations. They are genes with a mutation frequency significantly higher than the background mutation rate (BMR). We used Fisher’s exact test to screen for SMGs. The genes with more mutations are NUDT4B, HRNR, OBSCN, ATAD3B, and NBPF20. In combination with Fisher’s test, a P value of <0.05 is examined statistically significant. Therefore, we finally selected the ATAD3B gene (Table 6). As a gene with a high mutation frequency in tumor samples, it can serve as a target for gene therapy of CRC for further research and verification.

**TABLE 6:** High frequency mutation gene.

Gene	Muts	Total_Muts	Sample_affected	Sample_percent (%)	Pvalue
ATAD3B	2	4,038	2	7.69%	0.02098
CCDC27	1	4,038	1	3.85%	0.19955
AJAP1	1	4,038	1	3.85%	0.13003
STPG1	1	4,038	1	3.85%	0.13626
INPP5B	1	4,038	1	3.85%	0.30121
CYP4X1	1	4,038	1	3.85%	0.15893
LRRC8C	1	4,038	1	3.85%	0.24077
EXTL2	1	4,038	1	3.85%	0.10838
NBPF20	2	4,038	2	7.69%	0.57100
PPIAL4H	1	4,038	1	3.85%	0.05415
NUDT4B	5	4,038	5	19.23%	0.32450
HRNR	3	4,038	3	11.54%	0.07315
THBS3	2	4,038	1	3.85%	0.04261
FCRL5	1	4,038	1	3.85%	0.29308
ASTN1	1	4,038	1	3.85%	0.35835
KIF21B	1	4,038	1	3.85%	0.43000
ADIPOR1	1	4,038	1	3.85%	0.11962
PIK3C2B	1	4,038	1	3.85%	0.45588
OBSCN	4	4,038	4	15.38%	0.40588

Muts: The number of mutations in the gene; Total_Muts: The total number of mutation sites in the region was effectively analyzed; Sample_affected: The number of samples with mutations on the gene; Sample_percent: The proportion of the samples with mutations in this gene to the total sample number; Pvalue: The P-value calculated by Fisher’s Exact Test method.

### 3.7 Susceptibility gene screening

Cancer-predisposing genes (CPGs) are genes that may encode genetic diseases or acquired susceptibility to disease under environmental stimuli. Mutations that occur in an individual’s germ cells do not necessarily lead directly to cancer, but they can significantly increase an individual’s risk of developing cancer. We screened primarily for germ line mutations derived from sequencing 26 cases of normal tissue adjacent to cancer to screen for possible cancer predisposition genes. We screened for six candidate cancer susceptibility genes, where hom_ref indicates no mutation, het indicates a heterozygous mutation, and hom_alt indicates a homozygous mutation. The probability of these six genes to be mutated in normal tissue adjacent to cancer is CPA6 (3.85%), ZNF888 (46.15%), SH3BP1 (76.92%), ANKRD16 (30.77%), ATN1 (11.54%), and C4orf54 (80.77%). Take into account this, we roughly concluded that the more ideal cancer susceptibility genes are SH3BP1 and C4orf54 (Table 7). Since the probability of mutations in these two genes in normal tissues adjacent to cancer is relatively high, these two genes may be potential genetic targets for colorectal cancer and can be further verified.

**TABLE 7:** Susceptibility gene screening.

Samples/genes	CPA6	ZNF888	SH3BP1	ANKRD16	ATN1	C4orf54
A2	het	het	hom_ref	hom_ref	het	hom_ref
G2	hom_ref	hom_ref	het	het	hom_ref	het
B2	hom_ref	het	het	hom_ref	hom_ref	het
D2	hom_ref	hom_ref	hom_ref	hom_ref	hom_ref	het
F2	hom_ref	hom_ref	het	hom_ref	hom_ref	het
I2	hom_ref	het	hom_ref	hom_ref	hom_ref	het
K2	hom_ref	het	hom_ref	het	hom_ref	het
L2	hom_ref	het	het	hom_ref	hom_ref	het
E2	hom_ref	hom_ref	hom_alt	hom_ref	hom_ref	hom_alt
M2	hom_ref	hom_ref	het	hom_ref	hom_ref	hom_alt
N2	hom_ref	het	het	het	hom_ref	hom_alt
O2	hom_ref	het	hom_alt	hom_alt	hom_ref	het
P2	hom_ref	hom_ref	het	hom_ref	hom_ref	hom_alt
Q2	hom_ref	hom_ref	het	hom_ref	hom_ref	hom_ref
R2	hom_ref	hom_ref	het	hom_ref	hom_ref	het
S2	hom_ref	hom_ref	hom_alt	hom_alt	hom_ref	het
V2	hom_ref	het	hom_ref	hom_ref	hom_ref	het
X2	hom_ref	het	het	hom_ref	het	het
Y2	hom_ref	hom_alt	het	hom_ref	hom_ref	hom_ref
Z2	hom_ref	hom_ref	het	het	hom_ref	hom_ref
AA2	hom_ref	het	het	het	hom_ref	het
AB2	hom_ref	hom_ref	hom_alt	hom_ref	hom_ref	het
AC2	hom_ref	hom_ref	hom_ref	hom_ref	hom_alt	hom_ref
AD2	hom_ref	hom_ref	hom_alt	hom_ref	hom_ref	het
AE2	hom_ref	hom_ref	hom_alt	hom_ref	hom_ref	hom_alt
AF2	hom_ref	hom_alt	hom_alt	het	hom_ref	het

hom_ref means no mutation, het means heterozygous mutation, and hom_alt means pure and mutation.

### 3.8 Expression of SH3BP1 in pan-cancer, COAD, and READ

**FIGURE 5:**
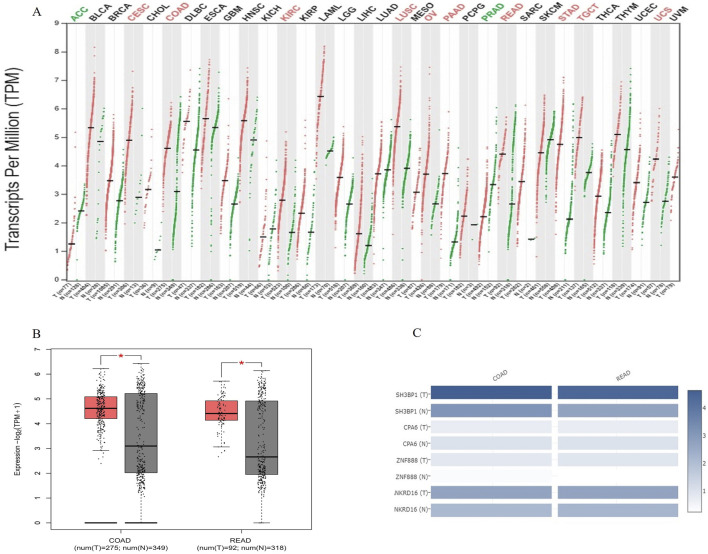
*SH3BP1* expression in pan-cancer, colon adenocarcinoma, and rectal adenocarcinoma. **(A)** A comparison of *SH3BP1* expression in 33 tumor (including COAD and READ) and normal tissues, based on data from the TCGA and GTEx databases. Red indicates tumor tissues and green indicates normal tissues. The cancer species name marked in red or green indicates that SH3BP1 is highly expressed in the corresponding tissue of that cancer species with P < 0.05. **(B)** Box plots showing differences in *SH3BP1* expression between COAD and READ tumor and normal tissues based on TCGA and GTEx databases. **(C)** A multi-gene comparison of *SH3BP1, CPA6, ZNF888*, and *NKRD16* expression levels in COAD and READ tumor and normal tissues based on whole-genome sequencing results and TCGA and GTEx databases.

A pan-cancer expression analysis revealed that *SH3BP1* is highly expressed in ten cancer types: Cervical Squamous Cell Carcinoma and Intracervical Adenocarcinoma (CESC), Colon Adenocarcinoma (COAD), Renal Clear Cell Carcinoma (KIRC), Lung Squamous Cell Carcinoma (LUSC), Ovarian plasma cystadenocarcinoma (OV), pancreatic adenocarcinoma (PAAD), rectal adenocarcinoma (READ), gastric adenocarcinoma (STAD), testicular germ cell tumor (TGCT), and uterine sarcoma (UCS). Two other cancers, adrenocortical carcinoma (ACC) and prostate adenocarcinoma (PRAD), showed reduced expression. Two other cancers, adrenocortical carcinoma (ACC) and prostate adenocarcinoma (PRAD), showed decreased expression (Figure 5A). Comparing the expression of *SH3BP1, CPA6, ZNF888*, and *NKRD16* in COAD and READ tumor and normal tissues revealed that SH3BP1 expression increased in COAD and READ and was more prevalent in tumor tissues than in the other susceptibility genes obtained by whole-genome sequencing (Figures 5B,C). These findings suggest that *SH3BP1* is upregulated in various tumor tissues, including COAD and READ.

### 3.9 Differential expression of the SH3BP1 gene between tumor and normal tissues and verification of its associated function

**FIGURE 6:**
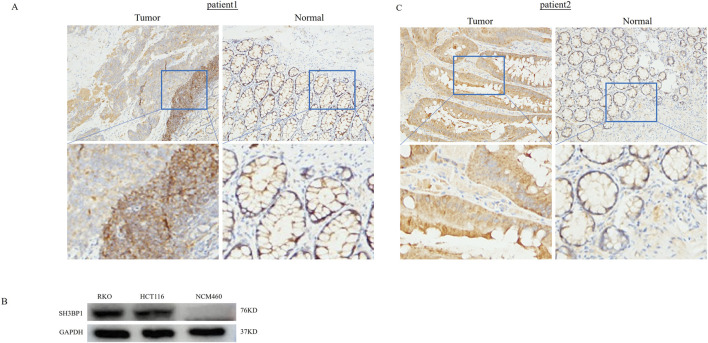
*SH3BP1* Expression Differs Between Cancerous and Normal Para-Cancerous Tissues. **(A,C)** Immunohistochemical staining of colorectal cancer patient tumors and para-cancerous specimens shows a clear difference in *SH3BP1* expression between the two. **(B)** Western blot analysis shows that *SH3BP1* is expressed at higher levels in colorectal cancer cells RKO and HCT116 than in normal intestinal epithelial cells NCM460.

**FIGURE 7:**
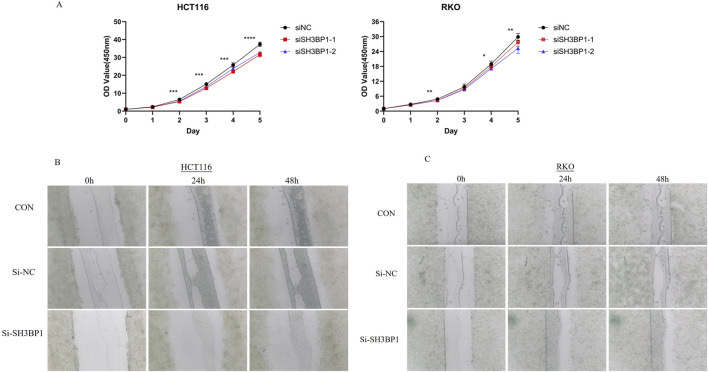
*SH3BP1* cell viability and scratch assays. **(A)** Cell proliferation ability was assessed using a cell viability assay (CCK8). *SH3BP1* knockdown cells proliferated at a slower rate than the si-NC group. **(B,C)** The scratch wound healing assay was used to determine the lateral migration ability of cells. This assay used si-NC and si-*SH3BP1*-transfected HCT116 and RKO cells, as well as untreated HCT116 and RKO cells from the CON group. There was a more significant difference in the migration rate of *SH3BP1* knockdown cells compared to the CON and si-NC groups.

Built on the genetic information obtained from susceptibility screening, we selected *SH3BP1* as the target gene for experimental verification. To investigate whether *SH3BP1* is differentially expressed in cancer and adjacent normal tissues, we performed protein blotting (WB) and immunohistochemistry (IHC) experiments. Our immunohistochemical analysis of pathological tissues from two patient groups revealed that *SH3BP1* expression was consistently higher in cancer tissues than in normal tissues (Figures 6A,C). Conversely, *SH3BP1* expression was negligible in normal human intestinal epithelial cells (NCM460) but significantly increased in RKO and HCT116 colorectal cancer cells (Figure 6B). These results imply that *SH3BP1* is differentially expressed in tumors and normal tissues adjacent to cancer. To further explore *SH3BP1*’s potential role in tumors, we downregulated the *SH3BP1* gene in HCT116 and RKO colorectal cancer cells by transfecting them with si-NC and si-*SH3BP1*, respectively. As shown in Figure 7A, the proliferative capacity of the colorectal cancer cells (HCT116 and RKO) was significantly reduced in the SH3BP1 knockdown group (si-*SH3BP1*-1, si-*SH3BP1*-2) compared to the si-NC group, as demonstrated by a cell viability assay (CCK8). Additionally, the migratory effect of *SH3BP1* on colorectal cancer cells was evaluated using a scratch wound healing assay. This assay showed that the migratory ability of colorectal cancer cells in the si-SH3BP1 group decreased, while the migratory ability of colorectal cancer cells in the si-NC group did not differ significantly from that of the CON group (Figures 7B,C). Thus, *SH3BP1* may promote tumor proliferation and migration in colorectal cancer and has the potential to be a new gene therapy target.

## 4 Discussion

Colorectal cancer is currently one of the tumors with high morbidity and mortality. Although colorectal cancer mortality can be reduced through screening, and surgical removal of the tumor can reduce the incidence of the disease (Gupta, 2022), the prevalence of early-onset colorectal cancer, which tends to develop in young people, will increase every year over the next decade. Approximately 30% of patients with early-onset CRC carry mutations that result in hereditary cancer susceptibility syndromes, and another 20% of patients have familial CRC (Mauri et al., 2019). Therefore, it is particularly important to discover and identify CRC genomes and susceptibility genes. To explore the genome of colorectal cancer, this study applied whole genome sequencing to the genome of colorectal cancer. Whole genome sequencing is widely used in human (Glanzmann et al., 2021), plasma (Wan et al., 2022), viruses (Thabet et al., 2023), microorganisms (Purushothaman et al., 2022), and so on. Whole genome sequencing can also detect the whole genome of tumors, such as gallbladder cancer (Okawa et al., 2023), prostate cancer (Liang et al., 2020), etc. Whole genome sequencing can also be used to sequence colorectal cancer, which can identify oncogenic mutated genes and signaling pathways, including oncogenes such as APC, tumor protein P53 (Tp53), and KRAS proto-oncogenes, and oncogenic pathways such as Wnt, P53, and phosphoinositide 3-kinase (Teng et al., 2018). This is the theoretical basis for its use in mapping the colorectal cancer genome.

This study included a total of 52 samples from colorectal cancer patients, of which 26 were colorectal cancer tissue samples and the other 26 were normal tissues adjacent to the cancer. These samples were subjected to whole genome sequencing (WGS) to comprehensively analyze the genome. Somatic and germline single nucleotide mutations, insertions, and deletions were detected, and it was determined that there is a significant difference in genomic bases between normal adjacent tissue and cancer tissue. This revealed the base mutations that may occur during the transformation of adjacent tissue into cancerous tissue, laying the foundation for further understanding in the future. On this basis, the mutation spectrum and mutation characteristics of tumor samples and high-frequency copy number mutation analysis were further obtained, and the tumor genome was further mined. As a result, we constructed the genomic landscape of colorectal cancer from base sequences to gene fragments through whole genome sequencing. Previous whole genome sequencing of colorectal cancer mostly focused on one of the colorectal cancer genomes, or did not have experimental verification, or only described the genome map of colorectal cancer or normal tissues. For example, Brannon et al. (2014) investigated the genomic differences between primary and metastatic colorectal cancer, while Schubert et al. (2020) focused on the significance of MSH6 and MUTYH genes in familial colorectal cancer, and Lee-Six et al. (2019) detailed the mutations in the epithelial cells of normal colorectal cancer. In contrast, this study not only provides a more comprehensive account of the major contents of the colorectal cancer genome but also shows the genome of the normal tissue adjacent to the cancer, thus clarifying the changes in the genome during the development of the cancer adjacent to the tumor. High-frequency mutated genes and disease susceptibility genes were identified in colorectal cancer tumors and adjacent normal tissues. Based on the susceptibility-to-disease genes, related genes were identified and experimentally verified, providing a theoretical and practical basis for the search of potential therapeutic targets for colorectal cancer.

We used whole genome sequencing to capture information on gene targets that have mutations in the genomes of colorectal cancer and adjacent cancers and analyzed and screened high-frequency mutated genes (*ATAD3B*) and susceptibility genes (*SH3BP1*, *C4orf54*). Whole genome sequencing revealed that *ATAD3B* has a mutation frequency of 7.69% in tumor samples (Table 5). Previous studies have found that when the expression of the tumor suppressor gene circ_*ATAD3B* is increased, it promotes the expression of miR-570-3p and inhibits the expression of *MX2*, thereby inhibiting the proliferation of breast cancer (Song et al., 2023). In addition, the higher the expression level of *ATAD3B* in hepatocellular carcinoma compared with normal liver tissue, the lower the survival rate of patients (Liu et al., 2019). This indicates that the gene has different expression levels in cancerous and normal tissues, which is consistent with our research results. It is also worth noting that the role of this gene may be different in different cancers, and there are currently no reports of *ATAD3B* gene research in colorectal cancer, so this gene can be used as a target gene for further research. The gene involved in this study, *SH3BP1*, has been found to be closely associated with various cancers in previous research reports. For example, in melanoma, overexpression of *SH3BP1* promotes the expression of Rac1 and Wave2, which promotes melanoma proliferation, invasion, and migration through the *SH3BP1/Rac1/Wave2* pathway (Sun et al., 2023). In leukemia, the *Cobbl1/SH3BP1/PACSIN2* axis promotes drug resistance and progression of chronic myeloid leukemia by regulating Rac1 activity, while the expression level of *SH3BP1* is inversely correlated with the prognosis of acute myeloid leukemia (Park et al., 2022; Yang et al., 2024). Notably, the function of *SH3BP1* in colorectal cancer remains unknown. Based on previous studies, we hypothesized that *SH3BP1* plays a tumor-promoting role in colorectal cancer. Consistent with our hypothesis, we first used pan-cancer species expression analysis and gene expression difference analysis in this study to verify differences in *SH3BP1* expression between tumor and normal tissues in various cancers, including COAD and READ. We then demonstrated differences in *SH3BP1* expression between colorectal cancer and normal tissues using protein blotting and immunohistochemistry experiments. Cell scratch and CCK8 experiments revealed that *SH3BP1* knockdown slows tumor proliferation and migration, suggesting that *SH3BP1* promotes proliferation and migration in colorectal cancer. As for *C4orf54*, it has only been found to be downregulated in recurrent inflammatory breast cancer (Qin et al., 2024) and may be associated with obstructive portal vein disease (Besmond et al., 2018). There is a relative lack of research on the rest of the gene, and it remains to be explored further. In conclusion, by performing whole genome sequencing of colorectal tumors and adjacent normal tissues, we have constructed a relatively complete genomic map, discovered genomic alterations from adjacent normal tissues to tumors, and screened potential targets for gene therapy. Our results provide a deeper and more reliable basis for genetic research and precision treatment of colorectal cancer.

There are also shortcomings in this article. First, the experimental verification is too simple, and there is still room for improvement. Wang (Wang et al., 2018) and others found that *SH3BP1* promotes the proliferation, migration, and chemoresistance of cervical cancer by affecting its downstream signaling pathway *Rac-Wave2*. In addition, Tao (Tao et al., 2016) and others also found that *SH3BP1/Rac/Wave2* can promote the production of vascular endothelial growth factor (VEGF) in hepatoma cells and induce the metastasis of liver cancer cells; Cicchetti (Cicchetti et al., 1995) and others also found that *SH3BP1* resists cell folding by acting on the downstream target Rac. Therefore, the experiment can further improve the validation of *SH3BP1/Rac/Wave2* pathway, whether it can also play the role of promoting proliferation, migration, metastasis, and chemotherapy resistance in colorectal cancer. Secondly, the validation of the functional mechanism is relatively immature. Xu et al. (2024) firstly found that tsRNA-GlyGCC was highly expressed in colorectal cancer by using biosignature analysis, which in combination with experimental validation indicated that tsRNA-GlyGCC could promote tumor progression and drug resistance; and Chen et al. (2024) also found that vitamin D receptor (VDR) expression was downregulated in colitis-associated colon carcinoma (CAC) tissues by using biosignature analysis. Another study by Chen et al. also used biosignature analysis to show that the expression of vitamin D receptor (VDR) was downregulated in colitis-associated colon cancer (CAC) tissues, and then it was verified that inhibition of VDR could delay the progression of CAC through *ex vivo* experiments. Thus, the functional mechanism study can be actively combined with the bioconfidence analysis to complete the chain of evidence, which can further support the experimental conclusions. Furthermore, the whole genome sequencing data of colorectal cancer is too single. Mendelaar et al. (2021) used whole genome sequencing to analyze the prognostic effect of metastatic colorectal cancer; Mendis et al. (2020) studied the combination of whole genome sequencing and circulating tumor DNA to detect the heterogeneity of colorectal cancer. Therefore, whole genome sequencing can continue to be used in the future to evaluate the prognosis or clinical treatment effect of colorectal cancer, which will help to improve the diagnosis and treatment of colorectal cancer in the clinic.

## 5 Conclusion

This study revealed significant differences in single nucleotide and insertion/deletion mutations between somatic and germ line cells, indicating substantial changes in paraneoplastic normal tissue genomes during cancerous transformation, particularly in non-coding regions. At the same time, analysis of copy number variations showed that samples exhibited different gene fragment alterations at different chromosomal loci. This favors the idea that genomic alterations play an essential role in tumorigenesis and development. These findings serve as the basis for analyzing tumor gene targets using whole genome sequencing. Additionally, the study found that the samples were dominated primarily by C>T mutation types, followed by T>C. C>A, T>A, T>G, and C>G occurred in decreasing proportions. When the mutation types were categorized more carefully into 96 types, the specific mutation types were dominated by C-G, C-T, G-A, G-C, and G-G. According to the distribution of these 96 types of mutations in each sample, the mutation characteristics of all samples were classified as either feature A or signature B. signature A was characterized by predominantly G-A, G-G, G-C, and G-T mutations, while signature B was characterized by predominantly A-G and C-G mutations. Most samples were predominantly signature A. In this paper, we found that *SH3BP1* gene expression increased in various cancers through pan-cancer analysis. In COAD and READ cancers, *SH3BP1* expression was higher in tumor tissues than in normal tissues. A cell proliferation and migration assay verified that *SH3BP1* has a role in promoting colorectal cancer. Further research is required to understand its role and mechanism in colorectal cancer.

## Data Availability

The data presented in the study are deposited in the NCBI SRA repository, accession number PRJNA1279985.
