# Supplementary material for: Exploring potential therapeutic targets for colorectal tumors based on whole genome sequencing of colorectal tumors and paracancerous tissues
Source: Front Mol Biosci. 2025 Jul 4;12:1605117. doi: 10.3389/fmolb.2025.1605117 (PMC12270881; doi:10.3389/fmolb.2025.1605117)
Supplement: Supplementary file 1 [file Supplementaryfile1.zip › Supplementary Material/Supplementary Table S2 Statistics of Single Nucleotide Mutations in Noncoding Regions of Somatic Cells.docx]

**Supplementary Table S2 Statistics of Single Nucleotide Mutations in Noncoding Regions of Somatic Cells**

| samples | ncRNA_exonic | ncRNA_intronic | ncRNA_splicing |
| --- | --- | --- | --- |
| A1 | 103 | 1345 | 1 |
| G1 | 127 | 1246 | 0 |
| B1 | 120 | 1515 | 0 |
| D1 | 119 | 1659 | 1 |
| F1 | 182 | 2277 | 2 |
| I1 | 187 | 2234 | 0 |
| K1 | 132 | 2183 | 1 |
| L1 | 131 | 1835 | 2 |
| E1 | 522 | 7031 | 2 |
| M1 | 160 | 1770 | 1 |
| N1 | 104 | 1253 | 0 |
| O1 | 124 | 962 | 0 |
| P1 | 124 | 1393 | 1 |
| Q1 | 117 | 1426 | 0 |
| R1 | 4406 | 68455 | 23 |
| S1 | 151 | 2244 | 2 |
| V1 | 97 | 1287 | 0 |
| X1 | 119 | 1369 | 0 |
| Y1 | 129 | 2085 | 0 |
| Z1 | 181 | 2527 | 0 |
| AA1 | 148 | 1892 | 1 |
| AB1 | 150 | 1991 | 1 |
| AC1 | 186 | 2420 | 2 |
| AD1 | 126 | 1661 | 0 |
| AE1 | 151 | 1896 | 0 |
| AF1 | 310 | 3325 | 5 |

ncRNA_exonic :The number of mutations occurring in the exon region of non-coding RNA; ncRNA_intronic: The number of mutations occurring in the intron region of non-coding RNA; ncRNA_splicing: The number of mutations occurring in the region of non-coding RNA splicing sites.
