# Supplementary material for: Exploring potential therapeutic targets for colorectal tumors based on whole genome sequencing of colorectal tumors and paracancerous tissues
Source: Front Mol Biosci. 2025 Jul 4;12:1605117. doi: 10.3389/fmolb.2025.1605117 (PMC12270881; doi:10.3389/fmolb.2025.1605117)
Supplement: Supplementary file 1 [file Supplementaryfile1.zip › Supplementary Material/Supplementary Table S3 Statistics of insertional deletion mutations in non-coding regions in somatic cells.docx]

**Supplementary Table S3 Statistics of insertional deletion mutations in non-coding regions in somatic cells**

| samples | ncRNA_exonic | ncRNA_intronic | ncRNA_splicing |
| --- | --- | --- | --- |
| A1 | 25 | 414 | 0 |
| G1 | 10 | 330 | 0 |
| B1 | 14 | 300 | 1 |
| D1 | 12 | 380 | 0 |
| F1 | 18 | 483 | 0 |
| I1 | 23 | 511 | 0 |
| K1 | 20 | 373 | 0 |
| L1 | 20 | 404 | 0 |
| E1 | 337 | 10295 | 3 |
| M1 | 27 | 489 | 0 |
| N1 | 18 | 437 | 0 |
| O1 | 12 | 270 | 0 |
| P1 | 14 | 452 | 0 |
| Q1 | 18 | 359 | 0 |
| R1 | 362 | 8022 | 3 |
| S1 | 29 | 804 | 1 |
| V1 | 18 | 416 | 0 |
| X1 | 10 | 389 | 0 |
| Y1 | 18 | 557 | 0 |
| Z1 | 28 | 677 | 0 |
| AA1 | 21 | 462 | 0 |
| AB1 | 30 | 413 | 0 |
| AC1 | 27 | 552 | 0 |
| AD1 | 19 | 390 | 0 |
| AE1 | 14 | 457 | 0 |
| AF1 | 20 | 380 | 1 |

ncRNA_exonic: The number of mutations occurring in the exon region of non-coding RNA; ncRNA_intronic: The number of mutations occurring in the intron region of non-coding RNA; ncRNA_splicing: The number of mutations occurring in the region of non-coding RNA splicing sites.
