# Supplementary material for: Exploring potential therapeutic targets for colorectal tumors based on whole genome sequencing of colorectal tumors and paracancerous tissues
Source: Front Mol Biosci. 2025 Jul 4;12:1605117. doi: 10.3389/fmolb.2025.1605117 (PMC12270881; doi:10.3389/fmolb.2025.1605117)
Supplement: Supplementary file 1 [file Supplementaryfile1.zip › Supplementary Material/Supplementary Table S4 Statistics of Single Nucleotide Mutations in Noncoding Regions of Germline Cells.docx]

**Supplementary Table S4 Statistics of Single Nucleotide Mutations in Noncoding Regions of Germline Cells**

| samples | ncRNA_exonic | ncRNA_intronic | ncRNA_splicing |
| --- | --- | --- | --- |
| A2 | 14031 | 221755 | 78 |
| G2 | 13924 | 215027 | 73 |
| B2 | 14222 | 230988 | 77 |
| D2 | 14367 | 234501 | 77 |
| F2 | 14222 | 230685 | 75 |
| I2 | 14386 | 230181 | 75 |
| K2 | 14353 | 231257 | 77 |
| L2 | 14262 | 230089 | 73 |
| E2 | 13923 | 228568 | 79 |
| M2 | 14261 | 228747 | 69 |
| N2 | 14357 | 231823 | 76 |
| O2 | 14575 | 230925 | 75 |
| P2 | 14153 | 230237 | 74 |
| Q2 | 14296 | 227515 | 76 |
| R2 | 14312 | 231956 | 71 |
| S2 | 14186 | 230501 | 71 |
| V2 | 13958 | 225763 | 61 |
| X2 | 14301 | 230530 | 74 |
| Y2 | 14371 | 229035 | 82 |
| Z2 | 14358 | 230158 | 73 |
| AA2 | 14380 | 229697 | 84 |
| AB2 | 14000 | 227230 | 68 |
| AC2 | 14235 | 229416 | 71 |
| AD2 | 14529 | 229184 | 76 |
| AE2 | 14090 | 229516 | 68 |
| AF2 | 14390 | 231208 | 72 |

ncRNA_exonic: The number of mutations occurring in the exon region of non-coding RNA; ncRNA_intronic: The number of mutations occurring in the intron region of non-coding RNA; ncRNA_splicing: The number of mutations occurring in the region of non-coding RNA splicing sites.
