# Supplementary material for: Exploring potential therapeutic targets for colorectal tumors based on whole genome sequencing of colorectal tumors and paracancerous tissues
Source: Front Mol Biosci. 2025 Jul 4;12:1605117. doi: 10.3389/fmolb.2025.1605117 (PMC12270881; doi:10.3389/fmolb.2025.1605117)
Supplement: Supplementary file 1 [file Supplementaryfile1.zip › Supplementary Material/Supplementary Table S5 Statistics of Insertion Deletion Mutations in Noncoding Regions of Germline Cells.docx]

**Supplementary Table S5 Statistics of Insertion Deletion Mutations in Noncoding Regions of Germline Cells**

| samples | ncRNA_exonic | ncRNA_intronic | ncRNA_splicing |
| --- | --- | --- | --- |
| A2 | 2085 | 55912 | 14 |
| G2 | 2089 | 55004 | 15 |
| B2 | 2235 | 63697 | 20 |
| D2 | 2281 | 64431 | 17 |
| F2 | 2207 | 63080 | 21 |
| I2 | 2260 | 61455 | 15 |
| K2 | 2228 | 62221 | 10 |
| L2 | 2198 | 62003 | 17 |
| E2 | 2093 | 59246 | 16 |
| M2 | 2241 | 61384 | 17 |
| N2 | 2215 | 60416 | 21 |
| O2 | 2254 | 62492 | 19 |
| P2 | 2144 | 58972 | 17 |
| Q2 | 2147 | 59299 | 12 |
| R2 | 2203 | 61270 | 12 |
| S2 | 2214 | 60602 | 14 |
| V2 | 2134 | 60102 | 14 |
| X2 | 2249 | 60689 | 22 |
| Y2 | 2304 | 63252 | 18 |
| Z2 | 2210 | 61377 | 16 |
| AA2 | 2231 | 60795 | 20 |
| AB2 | 2135 | 59297 | 18 |
| AC2 | 2207 | 60842 | 16 |
| AD2 | 2210 | 60261 | 18 |
| AE2 | 2149 | 61018 | 11 |
| AF2 | 2277 | 61197 | 15 |

ncRNA_exonic: The number of mutations occurring in the exon region of non-coding RNA; ncRNA_intronic: The number of mutations occurring in the intron region of non-coding RNA; ncRNA_splicing: The number of mutations occurring in the region of non-coding RNA splicing sites.
